# Evaluation of Data Quality of Four New Population Based Cancer Registries (PBCRs) in Chandigarh and Punjab, North India- A Quality Control Study

**DOI:** 10.31557/apjcp.2021.22.5.1421

**Published:** 2021-05

**Authors:** Md Abu Bashar, Jarnail S Thakur, Atul Budukh

**Affiliations:** 1 *Department of Community Medicine, Institute of Medical Sciences, BHU, Varanasi” and of Atul Budukh as “Homi Bhabha National Institute, Tata Memorial Centre, Mumbai, India. *; 2 *Community Medicine and SPH, PGIMER, Chandigarh, India. *; 3 *Tata Memorial Hospital, India. *

**Keywords:** PBCRs, IARC, comparability, validity, completeness

## Abstract

**Background:**

Population based Cancer Registries(PBCRs) are hallmark of cancer surveillance and cancer control activity .The value of cancer registries rely heavily on underlying quality of their data. Current study assessed data quality of four new PBCRs of Chandigarh, SAS Nagar, Mansa and Sangrur covering a total population of 4.5 millions on three quality parameters i.e. comparability, validity and completeness as recommended by International Agency of Research on Cancer(IARC), Lyon, France.

**Methods:**

For assessing comparability, data of the registries were reviewed in terms of system of classification and coding, definition of incidence date and rule for multiple primaries. For assessing validity (Accuracy) four different methods i.e. re-abstraction and re-coding, percentage morphologically verified cases (MV%), percentage of death certificate only (DCO%) cases and percentage of cases with other and unspecified sites (O and U%) were used. For assessing completeness of coverage, different semi-quantitative methods were used.

**Results:**

Re-abstraction done for 10% of the total incident cases yielded overall percentage agreement of 97.4%, 97.2%, 95.4% and 94.9% for PBCR Chandigarh, SAS Nagar, Mansa and Sangrur respectively. MV% was found to be 96.3% for PBCR Chandigarh, 92.8% for PBCR SAS Nagar , 89.3% for PBCR Mansa and 82.9% for PBCR Sangrur. Percentage of DCO cases and O and U cases were 1.4% and 2.8% for PBCR Chandigarh, 3.9% and 5.3% for SAS Nagar, 6.4% and 16.4% for Mansa and 6.3% and 8.3% for Sangrur. Completeness assessed through the various methods showed good level of completeness at PBCR Chandigarh and SAS Nagar and somewhat lower but acceptable level of completeness at PBCR Mansa and Sangrur.

**Conclusions:**

All the four PBCRs are comparable internationally. PBCR Chandigarh and SAS Nagar, predominantly urban registries, have higher accuracy of their data and good completeness levels as compared to predominantly rural registries of Mansa and Sangrur. Cancer estimates given by all the four registries are reliable and data from these registries can be utilized for planning cancer prevention and control activities in the region.

## Introduction

About 14 million new cancer cases and eight million cancer deaths are occurring annually around the globe and projections of cancer burden are alarming, with a predicted increase of 57% in the next two decades (Stewart and Christopher, 2014). The forecasted changes in the population demographics in the next two decades mean that even if current global cancer rates remain unchanged, the estimated incidence will rise to 21.4 million by 2030, with about two thirds of all cancer diagnoses occurring in low- and middle-income countries only (IARC, 2011). 

Having continuous, robust, and unbiased population data on cancer occurrence is necessary to monitor the impact of the disease, to build public health priorities and to evaluate the efficacy of cancer control programs in the community. The primary aim of Population based Cancer Registries (PBCRs) is to provide such data by collecting individual information on all patients diagnosed with cancer in the general population. Over the last 20 years, the information collected by PBCR expanded and improved and their role widened into becoming an indispensable tool for planning cancer control activities (Parkin, 2006).Cancer registries are of pivotal importance not only in assessing the cancer burden but also in measuring the impact of interventions in cancer prevention and control (Armstrong, 1992). Given sufficient resources, the modern cancer registry is active in a number of cancer control areas, including epidemiological research on the causes of cancer, the monitoring and evaluation of screening programme, and follow up of cancer patients (Parkin, 2008; Bray et al., 2014).

The value of cancer registries and its ability to carry out cancer control activities rely heavily on underlying quality of its data and quality control procedures in place (Storm, 1996; Bray et al., 2009). A cancer registry is a source of information and unreliable information is worse than no information .Quality control procedures are, therefore, instituted to identify the areas and degree of imperfection and thus assist in interpretation of data (Skeet, 1991). The benefit of PBCRs to cancer control programs and epidemiological research can be realized only to the extent that data are of comparable, high quality standard (Silva, 1999).

With this background, the current study was planned to assess the data quality of four newly established PBCRs i.e. Chandigarh, SAS Nagar, Sangrur, and Mansa in North India using three out of the four quality control parameters defined by International Agency of Research on Cancer (IARC), a specialized agency on cancer research of World Health Organization (WHO). These three quality parameters were: comparability, completeness (the proportion of all incident cancer cases in a registry population that is included in the registry database) and validity (the proportion of cases in the database with a given characteristic, such as site or age, that truly has the attribute) (Bray et al., 2014). 

## Materials and Methods

This was a cross sectional study carried out from August, 2014 to November, 2015 at four PBCRs located in union territory of Chandigarh and three districts i.e. SAS Nagar, Mansa and Sangrur in the state of Punjab, Northwest India.

PBCR Chandigarh covers the whole population of Union Territory of Chandigarh. Union territory of Chandigarh was the first planned city of India and is joint capital of two neighbouring states of India, Haryana and Punjab. Chandigarh has 97.3% of its population as urban and remaining 2.7% as rural (Census, 2011). PBCR SAS Nagar covers the District SAS Nagar of Punjab, a state in North West of India. District SAS Nagar is situated adjacent to Chandigarh on its North West side and has 54.8% of the population as urban and 45.2% of population as rural (Census, 2011). PBCR Mansa and Sangrur covered two other districts of Punjab which are predominantly rural comprising 2.9% and 6% of total population of Punjab state. Mansa has 78.7% of its population as rural whereas Sangrur has 68.8% of its population as rural Census, 2011).

PBCR Chandigarh, SAS Nagar (Ajitgarh) and Sangrur are functional since 1st January 2013 and PBCR Mansa since 1st April, 2013. Data collection is mainly through active method in which the medical social workers visits the different possible sources of cancer cases and deaths like hospital, pathology labs, offices of birth and death registrars, hospices etc. and also make village visits in rural areas to detect and register new cancer cases and cancer deaths.

Three out of four data quality indicators as defined by IARC i.e. Comparability, Validity (Accuracy) and Completeness were assessed in the current study. Timeliness couldn’t be assessed as these registries were only one and half years old at the time of study and for assessing timeliness, we need a gap period of 24 months from the end of diagnosis year (Bray et al., 2014). 

Comparability was assessed by reviewing the registries in terms of system of classification and coding, definition of incident case and incidence date and rule adopted for handling multiple primaries by the registries.

*Validity(Accuracy) was assessed through four different methods*

*i) Re-abstraction and re-coding*

A sample of 10% of the total incident cases of diagnosis year 2013 registered with the four PBCRs, selected through simple random sampling technique by computer generated random numbers, was taken and re-abstracted from the original sources i.e. diagnostic and treatment centers from where the case was notified and got registered and also by contacting the patient/relatives through home visits where ever required (the cases where hospital records were unavailable or incomplete) to ensure completeness of details. The core proforma used for registration containing 10 essential data items as defined by McLennan et al., (1991) in the standard IARC Publication was completed afresh for the selected cases without reference to original abstracts available at the registry by the Principal investigator. The original and the re-abstracted data items were then compared to find similarities and differences and accuracy rates for each of 10 data items and overall, were calculated for each of the four registries. 

*ii) Percentage of Morphologically verified (MV%) cases*

Percentage of morphologically verified (MV%) cases for each site, sex wise and overall, were calculated by examining the original registration forms of all the incident cancer cases registered at each of the four registries. Then, MV% values were tabulated by site and sex wise. MV% of the four registries was then compared with MV% of other Indian PBCRs under National cancer Registry programme (NCRP, 2013).

*(iii) Percentage of Death Certificate only (DCO%) cases*

All the incident cases registered at the four PBCRs were examined from the registry database to find out those cases in which there was no information available apart from a death certificate citing cause of death due to cancer. Then, percentage of these Death Certificate Only (DCO%) cases was calculated for all the four registries. The DCO% of each of the registries was then compared with that of other established PBCRs in India.

*(iv) Percentage of cases with others and unspecified sites *(O and U%)/ Primary site unknown cases (PSU%)-Registry database was examined to identify all those cases in which the primary site was not known (PSU) or not clearly defined or mentioned as others or not mentioned at all (left blank) and percentages of all such cases were calculated for each of the four registries. Then, these were compared with Oand U% of other established registries in India.

*Completeness was assessed through following semi-quantitative methods*

*(i) Historic data methods- Two methods were used based on this principle*

• Comparison of incidence rates in similar/different population- Age standardized Incidence rates, sex wise, of the four PBCRs were compared with those of some other well-established PBCRs in India. Statistically significant differences in the observed values of the registries with those of other registries were flagged.

• Incidence rates of childhood cancers - Age-specific incidence rates of three childhood age-groups i.e. 0-4, 5-9, and 10-14 years of the four PBCRs were compared with an ‘expected’ standard range of values. The limiting values for lowest and highest deciles, published in Volume X of Cancer Incidence in Five Continents (Forman et al., 2014) were used for comparing with age-specific rates of the above three age-groups.

*(ii) Mortality: Incidence Ratios*

Mortality data on cancer by sex and site, for the period from 1st January 2013 to 31st December 2013, were obtained from the respective registries. Then mortality: incidence (M: I) ratios for each cancer site, sex wise, were computed and tabulated. Then, M: I ratios , sex wise and overall, were compared with standard mean values for the Indian region mentioned in IARC technical report (Bray et al., 2014) and some of the established PBCRs in India under the National Cancer Registry Programme (NCRP, 2013).

*(iii) Average number of sources per case*

Same cancer case may have more than one source as multiple sources are used to collect data on cancer cases in a cancer registry (Bray et al., 2014). The justification for using as many sources as possible is that it reduces the possibility of cancer diagnoses going unreported,thus increasing the completeness of the registry (Parkin and Bray, 2009). Registered cancer cases having one source, two sources, three sources and more than three sources were computed, tabulated and average were taken to calculate the average number of sources/incident case. 

*Ethical Considerations*

Institute Ethical Committee (IEC) of Postgraduate Institute of Medical Education and Research(PGIMER), Chandigarh (U.T.), India reviewed and approved the study protocol. It was mainly a record based study with no direct involvement of patients, However, in some cases, visit to patients’ home were made for re-abstraction exercise. A written informed consent from such patients/family member was then taken to view the clinical records and investigations or enquire from the family members. Permission to access the database of the four cancer registries was granted by the Principal investigators of the respective registries. 

## Results

A total of 2801 incident cancer cases of diagnosis year 2013 and 2982 incident cancer cases of diagnosis year 2014 were registered at the four registries, registry wise details of the cancer cases are mentioned in Table 1.

*Comparability*

The classification and coding of tumors sites (topography) and morphology at all the four PBCRs was based on International Classification of Diseases for Oncology, 3^rd^ Edition (ICD-0-3) published by World Heath Organization(WHO) (Fritz et al. 2000). Incident cases registered at all the four PBCRs comprised of malignant neoplasms only with 5th digit behavior code either 3 or 6 according to ICD-O-3. The rule for the registration of incidence date in all the four registries follows the algorithm introduced by MacLennan and coworkers in the standard IARC publication (MacLennan, 1991).

The recording of multiple primary tumours at all the four PBCRs complied with the recommendations by International agency for Research on cancer (IARC) and International Association of Cancer Registries (IACR) for handling of multiple primaries (Working group Report, 2005). 

*Validity (Accuracy)*

*(i) Re-abstraction*

A total of 83 and 76 incident cases of diagnosis year 2013 registered with PBCR Chandigarh and SAS Nagar respectively were re-abstracted for 10 essential or key data items (Table 2). The overall average accuracy rates were 96.8% and 96.3% for PBCR Chandigarh and SAS Nagar respectively.The average accuracy rates for patient’s demographic details i.e. name, age, sex and address were found to be 99.4% and 99.3% for PBCR Chandigarh and SAS Nagar respectively whereas the average accuracy rates for tumour details i.e. date of diagnosis, basis of diagnosis, primary site, histology and behavior were found to be 95.1% for Chandigarh and 94.2% for SAS Nagar respectively. The first source of information was found to be correct in all the cases for both PBCR Chandigarh and SAS Nagar (Table 2). 

For PBCR Mansa and Sangrur, a total of 38 and 55 incident cases were re-abstracted respectively. The overall average accuracy rates for PBCR Mansa and Sangrur were found to be 94.2% and 93.4 % respectively. The average accuracy rates for patient’s demographic details (name, age, sex and address) were found to be 99.3% for PBCR Mansa and 98.6% for PBCR Sangrur. However, the average accuracy rates for tumour details (i.e. date of diagnosis, basis of diagnosis, primary site, histology and behavior) were found to be 89.5% for PBCR Mansa and 89.1% for PBCR Sangrur (Table 2).

*(ii) Percentage Morphologically Verified (MV%) cases*

The MV% cases, all sites combined,in males, were found to be highest at PBCR Chandigarh where 95.8% of all cancer cases registered were morphologically i.e. Histologically/Microscopically, verified whereas it was lowest at PBCR Sangrur where only 72.0% of all registered cases were morphologically verified. Among females, PBCR Chandigarh again has the highest MV% i.e. 98.1% and PBCR Sangrur has the lowest MV% (73.8%). The MV% cases at all the four registries were found to be higher among females compared to males (Table 3(a) and 3(b)). Figure 2 shows the comparison of Overall MV% both sex combined in the four PBCRs with other Indian registries.

*(iii)Percentage of Death Certificate Only (DCO%) cases*

%DCO cases were 1.4%, 3.9%, 6.4% and 6.3% at PBCR Chandigarh, SAS Nagar (Ajitgarh), Mansa and Sangrur respectively. Figure 3 shows comparison of %DCO cases in the four PBCRs with other Indian registries under the NCRP. 

*(iv)Percentage of cases with Other and Unspecified sites (O and U%)/Primary site unknown (PSU%)*

The percentage of cases with other and unspecified sites including the primary site unknown (C80) cases were 2.8%,5.3%, 16.4% and 8.3% at PBCR Chandigarh, SAS Nagar, Mansa and Sangrur respectively. Male registered cases were having more other and unspecified sites compared to female in all the four registries (Table 4). The comparison of Oand U% of the four PBCRs with other Indian PBCRs is shown in Figure 4.

*Completeness*

Figure 5(a) and 5(b) shows the comparison of Age-adjusted incidence rates (AARs) in the four PBCRs, among males and females, with other Indian registries.

Childhood cancer rates in the age-groups of 0-4, 5-9 and 10-14 years, for boys and girls, at all the four PBCRs with comparison to the standard reference are shown in Table 5. 

Comparison of M:I ratios at the four PBCRs with other Indian registries are shown in Figure 6*. *On an average, PBCR Chandigarh (2.2) and S.A.S Nagar registered >2 sources per incident case registered whereas PBCR Mansa and Sangrur have 1.8 and 1.7 sources per incident case registered.

**Table 1:** Distribution of Incident Cancer Cases, Sex Wise, Registered at the four PBCRs of Diagnosis Year 2013 and 2014

PBCRs name and location	Incident cases of year 2013			Incident cases of year 2014		
	Males	Females	Total	Males	Females	Total
U.T. Chandigarh	406	427	833	386	516	902
SAS Nagar (Ajitgarh), Punjab	334	433	767	325	429	754
Mansa, Punjab	187	216	403	203	244	447
Sangrur, Punjab	378	420	798	413	466	879

**Figure 1:**
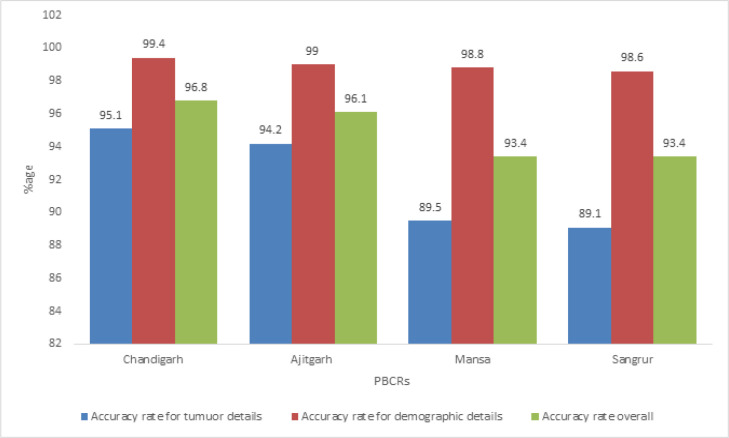
Accuracy Rates for Tumour Details, Demographic Details and Overall in Re-Abstraction Exercise of Selected Incident Cases Registered at the four PBCRs

**Table 2:** Results of Re-Abstraction of Selected Incident Cases of Diagnosis Year 2013 Registered at the four PBCRs

Sl. No.	Data items	PBCR Chandigarh		PBCR SAS Nagar		PBCR Mansa		PBCR Sangrur	
		No. in complete agreement(N=83)	%	No. in complete agreement (N=76)	%	No. in complete agreement (N=38)	%	No. in complete agreement (N=55)	%
1	Name	83	100	76	100	38	100.0	55	100
2	Age (in completed years)	82	98.8	74	97.4	37	97.3	53	96.3
3	Sex	83	100	76	100	38	100.0	55	100
4	Address/residence	82	98.8	75	98.6	38	100.0	54	98.8
5	Date of diagnosis	76	91.5	69	90.7	32	84.2	47	85.4
6	Basis of diagnosis	79	95.1	72	94.7	35	92.1	51	92.7
7	Primary site	78	93.9	70	92.1	34	89.4	49	89.1
8	Histology	76	91.5	68	89.4	31	81.5	45	81.8
9	Behavior	82	98.7	75	97.3	37	97.3	54	96.3
10	Source of information	83	100	76	100	38	100.0	54	96.3
	Overall	804/830	96.8	731/760	96.3	358/380	94.2	517/550	93.4

**Table 3 (a):** Percentages of Morphologically Verified (MV%) Cases, Site Wise, at the Four PBCRs, Male

Site	ICD-10	PBCR Chandigarh			PBCR SAS Nagar			PBCR Mansa			PBCR Sangrur		
		Cases	MV cases	MV %	Cases	MV cases	MV %	Cases	MV cases	MV %	Cases	MV cases	MV %
Mouth and Pharynx	C00-14	61	61	100	47	39	82.9	22	22	100	35	31	88.5
Other digestive organs	C15-21, 23-25	85	80	94.1	86	79	91.9	41	38	92.6	88	67	76.1
Liver	C22	8	10	80	13	8	61.5	5	4	80	15	7	46.6
Other respiratory organs	C30-32, 37-38	21	21	100	20	20	100	6	5	83.3	10	10	100
Trachea, Bronchus & Lung	C33,34	50	53	94.3	32	31	94.3	6	6	100	14	12	85.7
Bone	C40-41	4	3	75	4	4	100	3	2	66.7	5	3	80
Skin	C43-44	2	2	100	3	3	100	1	1	100	1	1	100
Mesothelial & soft tissues	C45-47, 49	2	2	100	3	3	100	2	1	50	5	5	100
Breast	C50	4	4	100	0	0	-	2	2	100	2	1	50
Other male genital organs	C60,62-63	5	5	100	3	3	100	9	9	100	9	8	88.8
Prostate	C61	32	32	100	29	28	96.5	6	5	83.3	24	22	91.7
Urinary organs	C64-68	23	22	95.7	20	19	95	4	3	75	13	9	69.2
Eye, brain & CNS	C69-72	13	12	92.3	12	8	75	8	5	62.5	22	12	54.5
Thyroid	C73-75	3	2	66.7	1	1	100	0	0	-	5	5	100
Non-Hodgkin’s lymphoma	C82-85,96	30	30	100	10	10	100	4	3	75	11	9	81.8
Other hematopoietic neoplasm	C81, 88-95	41	41	100	26	26	100	22	20	90.9	41	39	95.2
Other & unspecified cancers	C26,39,48,76-80	17	14	82.3	25	18	72	40	37	92.5	78	44	56.4
Total		406	389	95.8	334	306	91.6	179	159	88.8	378	272	72

**Table 3 (b):** Percentages of Morphologically Verified(MV%) Cases, Site Wise,at the Four PBCRs, Female

Site	ICD-10	PBCR Chandigarh			PBCR SAS Nagar			PBCR Mansa			PBCR Sangrur		
		Cases	MV cases	MV %	Cases	MV cases	MV %	Cases	MV cases	MV %	Cases	MV cases	MV %
Mouth and Pharynx	C00-14	12	12	100	17	17	100	5	5	100	11	11	100
Other digestive organs	C15-21, 23-25	56	56	100	63	57	90.5	39	36	93.2	62	52	83.8
Liver	C22	5	5	100	9	6	66.7	1	1	100	10	4	40
Other respiratory organs	C30-32, 37-38	4	4	100	4	3	75	0	0	-	3	3	100
Trachea, bronchus & lung	C33,34	16	16	100	11	10	90.9	2	1	50	1	1	100
Bone	C40-41	3	3	100	2	2	100	1	1	100	5	4	80
Skin	C43-44	1	1	100	2	2	100	1	1	100	0	0	-
Mesothelial & soft tissues	C45-47,49	1	0	0	3	3	100	3	3	100	0	1	0
Breast	C50	154	152	98.7	137	135	98.5	39	39	100	96	89	92.7
Other female genital organs	C51-52, 54-58	56	55	98.2	55	53	96.3	17	17	100	46	38	82.6
Cervix uteri	C53	43	43	100	58	55	94.8	48	47	97.9	77	48	62.3
Urinary organs	C64-68	9	9	100	7	7	100	9	8	88.9	6	2	33.3
Eye, brain & CNS	C69-72	6	7	85.7	10	9	90	2	2	100	13	7	53.8
Thyroid	C73-75	9	9	100	5	5	100	3	3	100	7	7	100
NHL	C82-85, 96	19	19	100	15	15	100	1	1	100	5	5	100
Other hematopoietic neoplasm	C81, 88-95	24	24	100	28	27	96.4	14	14	100	30	30	100
Others & unspecified cancers	C26,39,48,76-80	6	2	33.3	16	10	62.5	24	17	70.8	48	28	58.3
Total		427	419	98.1	433	411	95	209	194	92.8	420	310	73.8

**Table 4:** Percentage of Cases with Other and Unspecified (O & U%) Sites Registered at the Four PBCRs

PBCRs	Male			Female			Overall O & U%
	Total incident cases	Other & unspecified site cases (O & U)	O & U %	Total incident cases	Other & unspecified site (O & U)	O & U %	
Chandigarh	406	17	4.2	427	6	1.4	2.8
SAS Nagar	333	25	7.5	433	16	3.7	5.3
Mansa	179	42	22.5	209	23	10.6	16.4
Sangrur	360	37	10.2	409	27	6.6	8.3

**Figure 2:**
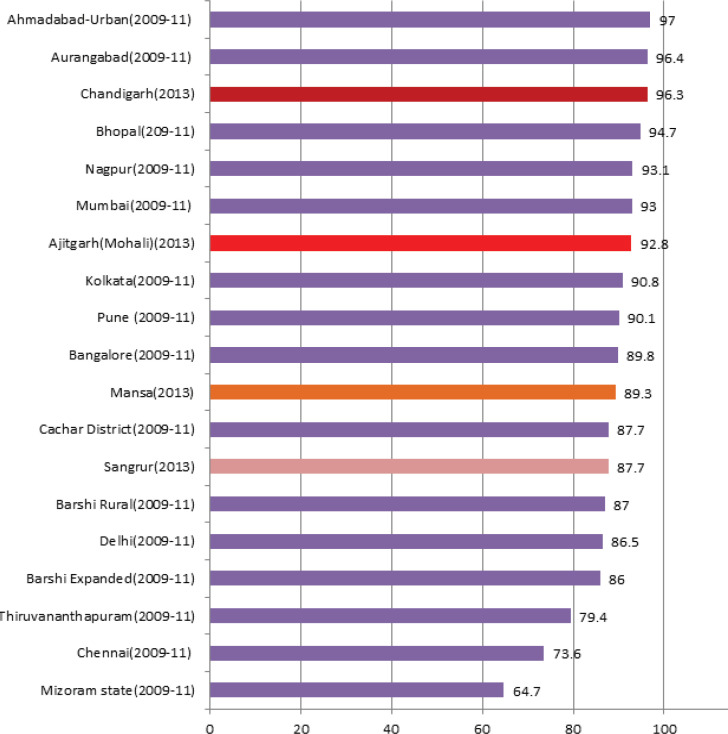
Comparison of Percentage of Cases Morphologically Verified (MV%) at the Four PBCRs with Other PBCRs in India (All Sites Combined)

**Figure 3:**
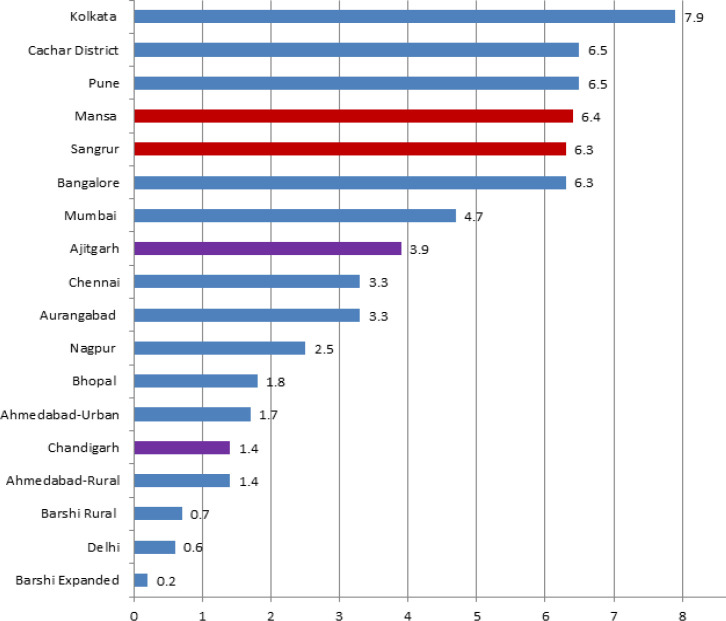
Comparison of Percentage of Death Certificate only (DCO%) Cases at the four PBCRs with Other Indian PBCRs

**Figure 4:**
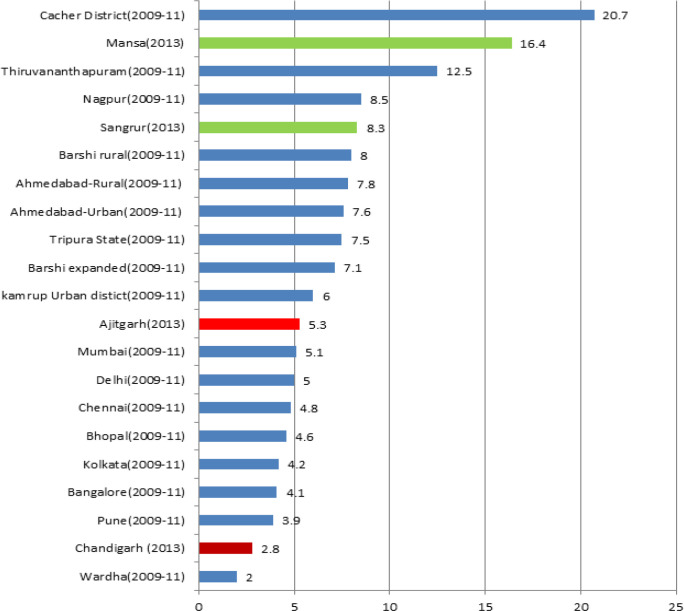
Comparison of Percentage of Cases with Other & Unspecified Sites (O & U%) at the four PBCRs with Other Indian PBCRs

**Figure 5 (a):**
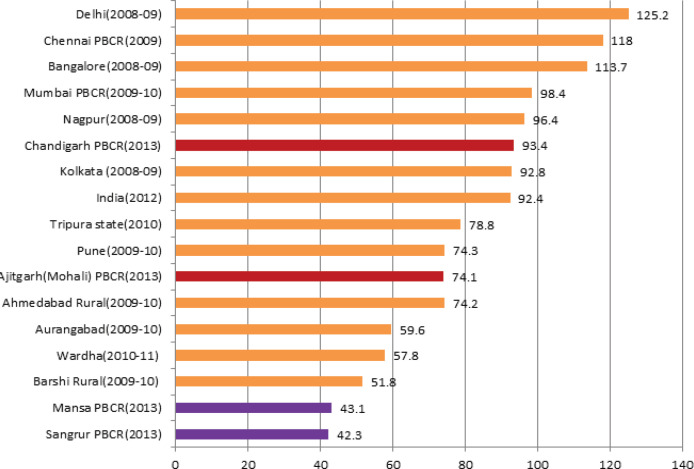
Comparison of Age-Adjusted Incidence Rates (AARs) at the Four PBCRs with Other Indian PBCRs, Male

**Figure 5(b):**
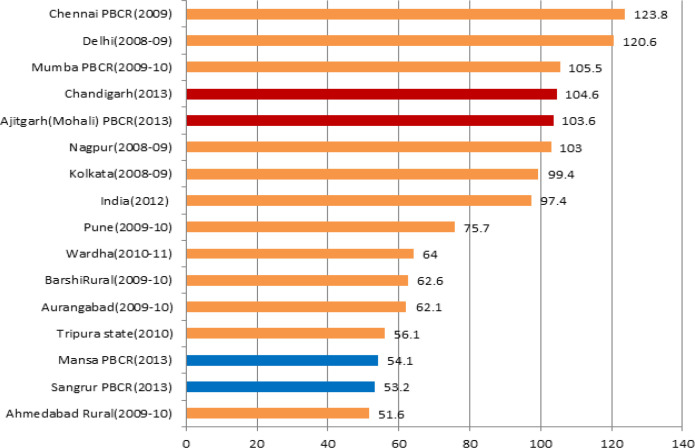
Comparison of Age-Adjusted Incidence Rates (AARs) at the Four PBCRs with Other Indian PBCRs, Female

**Figure 6:**
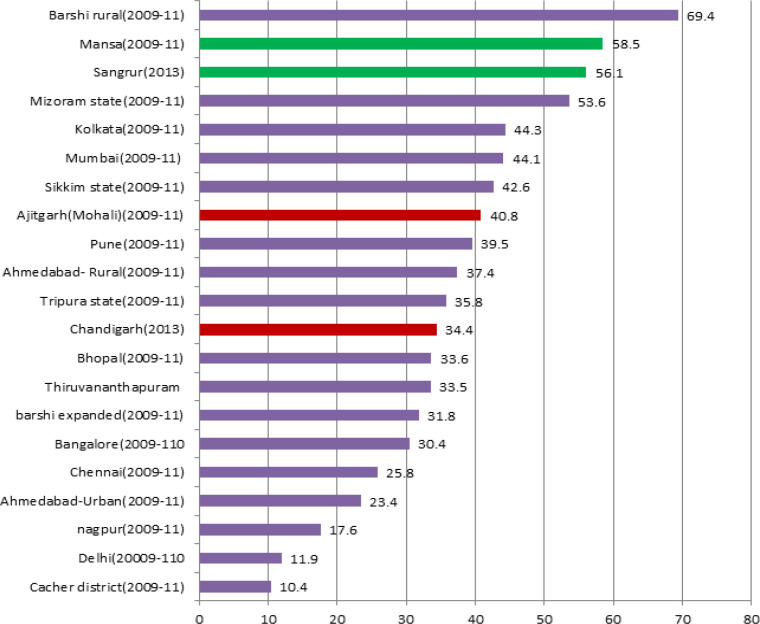
Comparison of Mortality to Incidence Ratio (M:I),Overall, of the Four PBCRs with Other Indian PBCRs

**Table 5:** Comparison of the Age Specific Incidence Rates (ASIR) Per Million for Childhood Cancers, by Gender, at the Four PBCRs with Standard Reference*

Age (in years)	Boys				Reference range*	Girls				Reference range*
	Chandigarh	SAS Nagar	Mansa	Sangrur		Chandigarh	SAS Nagar	Mansa	Sangrur	
0-4	6.8	7.6	12.8	1.4	12.3-24.7	5.1	14.3	0.0	1.4	9.7-21.4
5-9	10.0	9.3	2.9	3.7	8.5-15.6	11.8	5.6	0.0	1.6	6.9-12.0
10-14	3.7	7.4	4.8	1.1	8.5-15.0	4.8	3.4	6.2	4.3	6.8-13.6

*Cancer Incidence in Five Continents, Vol. X, IARC, Lyon,France

## Discussion

Current study was first of its kind from India which simultaneously assessed the four PBCRs on three out of the four data quality indicators set by IARC and IACR for PBCRs. An earlier study from Chennai,India assessed only completeness whereas a recent study from Assam assessed validity and completeness of the PBCR data (Gajalakshmi et al., 2001; Sharma et al., 2017). Quality control is the most important aspect of any cancer registry as the cancer estimates and burden given by the registry would be close to its true value only when there is an effective mechanism of quality control in the registry (Bray et al., 2009).

In our study, all the four PBCRs have coded all malignancies according to ICD-0-3 manual released in the year 2000 by IARC/WHO. Furthermore, all topographical sites, coded according to ICD-O-3, were entered into the canreg5 software developed by the IARC and were converted to ICD-10. All the four registries were found to be comparable to other established national and international registries.

The overall accuracy rates of 96.8% for PBCR Chandigarh and 96.3% for PBCR SAS Nagar in the re-abstraction exercise exceeded the Surveillance, Epidemiology, and End Results (SEER) Program goal of 95% accuracy rate for achieving a five star performance (Hofferkamp, 2008) implying high validity of data of these registries.Similarly, the overall accuracy rate of PBCR Mansa was 93.4% and that of PBCR Sangrur were 93.6% receiving a four star performance according to SEER standards (Hofferkamp, 2008). These findings imply that registry personnel were able to collect data with a high degree of accuracy.

The overall accuracy rates of these four registries were also comparable to PBCRs under National Cancer Registry Programme (NCRP) where overall accuracy rate in re-abstraction is reported to be around 95% with less than five percent error rates (NCRP, 2013). The re-abstraction audits are a part of routine quality control activity in these registries, which are done on yearly basis. However, the details are unknown. Our study is first of its kind from India where re-abstraction was performed independent of the registry implying high validity of our findings.

In a study from Netherlands, IKL cancer registry performed a study with the aim of comparing data supplied by clinicians with data collected by registration personnel.The percentage agreement levels for date of incidence, primary site, histologic type and behavior were 54%,79%,86% and 98% respectively which is lower than the agreement levels observed for the four registries in our study (Schouten et al.,1993). However, in a study to assess accuracy of Scottish cancer registry data, serious discrepancies were judged to have occurred in only 2.8% of the registered cases which is lower than the discrepancies seen at the four registries in our study (Brewster et al.,1994). In another study to assess Scottish cancer registration data, a random sample of 3500 primary cancers of year 1997 was re-abstracted. Reliability was high for demographic, diagnostic and facts of treatment details but less reliable for grade of differentiation, staging variables and dates for treatment similar to our findings (Brewster et al., 2002).

Percentage of Morphologically verified (MV%) cases at PBCR Chandigarh (96.3%) was found to be higher than some of the established urban registries in the country i.e. Mumbai (93%), Delhi (86.5%), Bhopal (94.7%), Nagpur (93.1%), Kolkata (90.8%) and Bangalore (89.8%) (NCRP, 2013) implying high data quality. MV% cases at PBCR SAS Nagar (92.8%) were also found comparable to these registries. MV% cases at PBCR Mansa and Sangrur were 89.3% and 87.7% respectively. As these registries cover regions which are predominantly rural (76% in Mansa and 69% in Sangrur as per Census, 2011), there are lack of diagnostic centers including pathology labs for histological verification of tumour and lack of cancer treatment facilities for referring the suspected cancer cases for confirmation. However, their MV% were found comparable to other established rural registries like Barshi Rural (87%), Barshi expanded (86%), Cachar district(87.7%) in the country (NCRP, 2013).PBCR Chandigarh has MV% cases comparable to some of the European and American registries implying high validity of its data and cancer registration (Larsen et al., 2009; Bray et al., 2017; National Cancer Registry, 2012) .

The gold standard for DCO% in some of the American registries (SEER and NACCR) is of less than 5% (Hofferkamp, 2008). However, “Cancer incidence in five continents”, Vol.-X , an official Publication by IARC on global cancer burden, pattern and distribution, accepted data from those PBCRs where the DCO% were less than 20% (Forman et al., 2014). The very low percentage of DCO cases at PBCR Chandigarh and SAS Nagar i.e. 1.4% and 3.9% respectively, reflects effective trace back mechanism of all death certificate notified (DCN) cases at these registries as most of the cancer death cases from this region take treatment at Chandigarh and SAS Nagar only owing to good diagnostic and treatment facilities available in this region. The DCO% at PBCR Chandigarh (1.4%) were found to be better than some of the established PBCRs in the country i.e. Mumbai (4.7%), Chennai (3.3%), Nagpur (2.5%) and Bhopal (1.8%) whereas DCO% cases of PBCR SAS Nagar (3.9%) was found comparable to these registries (NCRP, 2013). Both of these registries too qualify for the gold standard of DCO as per recommendations of some of the American registries (NAACCR) (Hofferkamp,2008).

However, the DCO% in PBCR Mansa and Sangrur were higher than PBCR Chandigarh and SAS Nagar. The reason attributed to this could be the lack of successful trace back of DCN cases owing to poor quality of records available at the hospitals in these regions and death cases taking treatment outside of these regions owing to lack of treatment facilities and absence of specialized cancer centers in these regions. Sometimes, patient’s family members and relatives burn the medical records of the patients after his/her death, making it difficult to know the details of the case. DCO cases for these regions were calculated by considering in mind the fact that some of the cancer death cases don’t have even death certificate and in that case, patient’s relative remarks were the only way to establish the death case as cancer. Out of 25 cases which were classified as DCO at PBCR Mansa, none of the patient’s relative had any records and the attempt to trace back these cases to the diagnostic and treatment centers were also unsuccessful. As certification of cause of death is still poor in rural areas of India, there’s need to include a separate category for such cases where death certificates of the cancer cases are not available and only patients relative’s remarks are available which could be utilized through verbal autopsy to ascertain the cause of death and even utilized further to know the primary site of the cancers (Sakaranaraya and Swaminathan, 2012). The DCO% of PBCR Mansa and Sangrur were found comparable to some of the established registries like Bangalore (6.3%), Pune (6.5%), Cacher district (6.5%) and Kolkata (7.9%) and lower than that of Thiruvananthapuram (8.9%), Kamrup Urban(9.8%), Mizoram (14.5%) and Dibrugarh district(14.8%) (NCRP, 2013).

The low percentage of Other and Unspecified site (O and U%) cases at PBCR Chandigarh (2.8%) and SAS Nagar (5.3%) was due to high percentage of Morphologically Verified (MV%) cases at these registries. Also, owing to better diagnostic and treatment facilities available in this region, most of the cancer patients of the region present in early stages leading to detection of the true primary site. The O and U% of Chandigarh (2.8%) were better than some of the established urban registries of India like Bangalore (4.1%), Kolkata (4.2%), Chennai (4.8%), Delhi (5.0%) and Mumbai (5.1%) (NCRP, 2013). O and U% of SAS Nagar was also found comparable to these registries and better than some other Indian urban registries like Kamrup Urban(6.0%), Barshi expanded(7.1%), Ahmedabad Urban(7.6%), Nagpur(8.5%) and Thiruvananthapuram (12.5%) (NCRP, 2013).

The higher number of Primary site unknown cases are clearly related to the quality of diagnostic information as well the poor documentation of the medical records (Bray and Parkin, 2009). The O and U% at PBCR Sangrur (8.3%) and PBCR Mansa (16.4%) were somewhat higher owing to lack of diagnostic centers in these regions and patients presenting at advanced stages making it difficult to ascertain the actual primary site. Further, high DCO% in PBCR Mansa and PBCR Sangrur means high O and U% as cases registered on basis of death certificates lacks the primary sites. However, the cut off value of having less than 20% of cases in this category for the registry to be included in IARC Publication“Cancer Incidence in Five Continent”, volume X (Forman et al., 2014) was fulfilled by all the four PBCRs. PBCR Sangrur and Mansa have comparable O and U% with other rural registries in India like Barshi Rural(8.0%), Sikkim state(8.6%), Mizoram state(11.9%) and Cachar district(20.7%) (NCRP, 2013)

The completeness of the registries i.e. extent to which all the cases of cancer incident in the population are included in the registry data base (Skeet, 1991; Parkin and Bray, 2009) were assessed in the current study by semi-quantitive methods because of lack of time and resources although more objective way is by using quantitative methods (Parkin and Bray, 2009). As there was no data from any registries available previously for the regions covered by the four PBCRs, the incidence rates of the four registries were compared with the incidence rates of other established registries from different regions in the country (NCRP, 2013). This method has been recommended by IARC for assessing completeness of any PBCR in its standard publication (Parkin and Bray;2009).The age adjusted incidence rates(AARs) of PBCR Chandigarh for males and females were 93.4 per 100, 000 and 104.6 per 100,000 population respectively. They were found comparable to the AARs of Mumbai (98.4 for males and 105.5 for females) (NCRP, 2013) and India (92.4 for males and 97.4 for females) estimated by GLOBOCAN (Ferlay et al., 2015) which implies good completeness of coverage at PBCR Chandigarh. Similarly, AARs of PBCR SAS Nagar for males was 74.1 per 100,000 population which was found comparable to Pune registry (AAR of 74.3 per 100,000) (NCRP, 2013) and somewhat lower than the Indian figure of 92.4 (Ferlay et al., 2015).This may indicate towards under-registration of male cancer cases by the registry or it may be due to actual lower incidence of cancer among males in SAS Nagar. The AAR for females at PBCR SAS Nagar was 103.6 per 100,000 which was found comparable to Mumbai, Nagpur and Kolkata registries and higher than the Indian figure of 97.4 for female by GLOBOCAN (Ferlay et al., 2015) implying good completeness for female cancer cases at the registry. 

Similarly, the AARs for males in PBCR Mansa and Sangrur were 43.1 and 42.1 per 100,000 male population respectively which were found comparable to the established rural registry of Barshi Rural (51.8 per 100,000) and somewhat lower than Wardha (57.8 per 100,000), Aurangabad (59.6 per 100,000) (NCRP,2013). For females, the AARs at PBCR Mansa and Sangrur (54.1 and 53.2 per 100,000 female population respectively) were somewhat lower than that of Barshi Rural (62.6 per 100,000) (NCRP, 2013) implying the possibility of under-registration of female cancer cases at these registries.

The incidence rate of cancers in childhood age-groups (0-4,5-9 and 10-14 years) shows much less variability than adults, although there are well-documented differences by geography or ethnicity for specific types of childhood cancers (Parkin and Bray, 2009). The incidence rates for the age group of 5-9 years at PBCR Chandigarh,both for boys and girls, fell in the expected range of childhood cancer rates as mentioned in the volume X of CI5 (Forman et al., 2014) indicating good completeness for this age-group where as in the age-groups of 0-4 and 10-14 years, it fell below the expected range indicating towards the possibility of under-registration. Similarly, for PBCR SAS Nagar, the incidence rates for the age- groups of 0-4 years, both in boys and girls and 5-9 years, in boys only, fell in expected range indicating completeness for these age-groups. For rest of the age-groups, it fell below the expected range. At PBCR Mansa, the incidence rate for the age-group of 0-4 years in boys only, fell in the expected range whereas for other age-groups in boys and all the age-groups in girls, it fell below the expected range. There was no case registered in the age-groups 0-4 and 5-9 years in girls indicating missing of cases by the registry. For PBCR Sangrur, the incidence rates in all age-groups, both for boys and girls, fell below the expected range pointing towards under-registration of childhood cancers by the registry.

Mortality: incidence (M:I) ratio, an important measure of completeness, is calculated by obtaining mortality data from a source independent of the registry (usually the vital statistics system) (Parkin and Bray,2009) but due to lack of reliable mortality data in developing countries like India, registries in India calculate the M:I ratio by obtaining the data on cancer mortality from the registries itself. The M: I ratio percentage of PBCR Chandigarh for males (40.4%) and females (28.8%) were found comparable to the mean standard values of M:I for India i.e. 40.2% for males and 32.8% for males (Bray et al., 2014)..For PBCR SAS Nagar, the M:I ratios of 53.2% for males and 33.2% for females were also found comparable to the mean standard values (Bray et al., 2014). Further, the overall M:I ratios of PBCR Chandigarh (34.4%) and SAS Nagar (40.8%) were found comparable to urban registries like Mumbai (44.1%), Bangalore(30.4%), Bhopal(33.6%) and Kolkata (44.3%) implying good completeness of these two registries. For PBCR Mansa and Sangrur, the M:I ratios were found somewhat higher than the mean standard values for India but as these standard values were calculated by taking average of only 9 established PBCRs under NCRP, eight of which were predominantly urban, they mainly reflect the M:I ratios of urban registries and hence cannot be used to compare with predominantly rural registries like Mansa and Sangrur. However, the overall M:I ratio of Mansa (58.5%) and Sangrur (56.1%) were found comparable to similar registries like Mizoram state (53.6%) and Barshi Rural (69.4) but lower than some other rural registries like Ahmedabad Rural (37.4%) and Wardha (44.1%) (NCRP,2013).Hence, there might be under-registration of incident cancer cases in these two registries. 

The average number of distinct sources per incident case of 2.2 and 2.1 at PBCR Chandigarh and SAS Nagar respectively gives an indication that very few cases were likely to be missed by these registries. The number of sources per case for these two registries is comparable to some of the European registries like Irish National Cancer Registry which had 2.8 sources per case (National Cancer Registry, 2012). PBCR Mansa and Sangrur have 1.8 and 1.7 sources per incident case which is quite good considering the rural background of these two registries.

In conclusion, all the four PBCRs are nationally and internationally comparable. Data on cancer cases at predominantly urban registries of PBCR Chandigarh and SAS Nagar have high validity and accuracy whereas that of predominantly rural registries of Mansa and Sangrur were also found to be of acceptable standards. The accuracy of data on demographic details in all the registries was found to be excellent but accuracy of data on tumuor details were somewhat lower and warrants improvement. The prevention of these errors asks for more attention in the training of the registry personnel on abstracting tumour details of the cancer cases. Similarly, high percentage of DCO and O & U cases at PBCRs Mansa and Sangrur is a major challenge and requires concerted efforts for improvement in subsequent years of registration. Completeness assessed through different semi-quantitative methods yielded good level of completeness in urban registries of PBCR Chandigarh and SAS Nagar. The predominantly urban registries of Chandigarh and SAS Nagar complies to most of the quality parameters and standards set by IARC and may qualify for inclusion in the series ‘Cancer Incidence in Five Continents’ published by IARC. Further, cancer estimates given by all the four registries are reliable and data from these registries can be utilized for planning cancer prevention and control activities in the region. 

## Author Contribution Statement

MAB and JST conceived the idea of the study, MAB done the literature review, data collection and performed data analysis, MAB wrote the first and subsequent drafts of the study. JST and AB revised the subsequent drafts. All authors approved the final draft of the paper for submission.

## Limitations

The values in the results may not coordinate with the exact values in the original PBCR reports. Because, while writing this paper in 2015, the tentative data was taken and included for data analysis. Whereas the original PBCR reports were updated later in year 2016. So, the values may not exactly match but they are approximately the same

## References

[B1] Armstrong BK (1992). The role of cancer registries in cancer control. Cancer Causes Control.

[B4] Bray F, Parkin DM (2009). Evaluation of data quality in the cancer registry: principles and methods Part I: comparability, validity and timeliness. Eur J Cancer.

[B5] Brewster D, Crichton J, Muir C (1994). How accurate are Scottish cancer registration data?. Br J Cancer.

[B6] Brewster DH, Stockton D, Harvey J, Mackay M (2002). Reliability of cancer registration data in Scotland, 1997. Eur J Cancer.

[B8] Ferlay J, Soerjomataram I, Dikshit R (2015). Cancer incidence and mortality worldwide: sources, methods and major patterns in GLOBOCAN 2012. Int J Cancer.

[B11] Gajalakshmi V, Shawaminathan R, Shanta V (2001). An independent survey to assess completeness of Registration: Population based Cancer Registry. Asian Pac J Cancer Prev.

[B13] Larsen IK, Småstuen M, Johannesen TB (2009). Data quality at the cancer registry of Norway: an overview of comparability, completeness, validity and timeliness. Eur J Cancer.

[B15] National Cancer Registry Programme (2013). Three-year Report of Population Based Cancer Registries: 2009-11, Report of 25 PBCRs; National Cancer Registry Programme, ICMR, Bangalore.

[B17] Parkin DM (2006). The evolution of the Population-based Cancer Registry. Nat Rev Cancer.

[B18] Parkin DM (2008). The role of cancer registries in cancer control. Int J Clin Oncol.

[B19] Parkin DM, Bray F (2009). Evaluation of data quality in the cancer registry: principles and methods Part II. Completeness. Eur J Cancer.

[B20] Hofferkamp J (2008). Standards for Cancer Registries Volume III: Standards for Completeness, Quality, Analysis, Management, Security and Confidentiality of Data.

[B21] Sakaranarayan R, Swaminathan R (2012). Verbal-autopsy-based projection of cancer deaths in India. Lancet.

[B22] Schouten LJ, Jager JJ, van den Brandt PA (1993). Quality of cancer registry data: a comparison of data provided by clinicians with those of registration personnel. Br J Cancer.

[B23] Sharma A, Sharma JD, Kataki AC (2017). Quality assessment and improvement of cancer registration system in Kamrup Urban District: A report. Indian J Cancer.

[B24] Silva IS, editor (1999). The role of cancer registries;In Cancer Epidemiology: Principles and Methods; International Agency for Research on Cancer.

[B25] Skeet RG, Jensen OM, Parkin DM, MacLennan R, Muir CS, Skeet RG (1991). Quality and quality control. Cancer Registration: Principles and Methods.

[B26] Stewart BW, Wild CP (2014). World Cancer Report 2014.

[B27] Storm HH (1996). Cancer registries in epidemiologic research. Cancer Causes Control.

[B28] Working Group Report (2005). International rules for multiple primary cancers (ICD-0 third edition). Eur J Cancer Prev.

